# Impact of psychological contract breach on firm’s innovative performance: A moderated mediation model

**DOI:** 10.3389/fpsyg.2022.970622

**Published:** 2022-08-24

**Authors:** Lizhe Zhang

**Affiliations:** Sichuan Administration College, Ministry of Social and Cultural Education and Research, Chengdu, China

**Keywords:** psychological contract breach, knowledge hiding, moral disengagement, firm innovative performance, perceived supervisor support

## Abstract

Organizations are seeking ways to be more competitive in the market. Globalization also paves the way for additional challenges for firms to compete in today’s knowledge-based economy and competitive corporate settings. The psychological contract breach (PCB) of employees could be a possible reason to slow down the firm’s innovative performance. Based on the social exchange theory, the present study assumes that a PCB negatively affects a firm’s innovative performance. The present study also assessed the mediating role of knowledge hiding (KH) and moral disengagement (MD) in the relationship between PCB and a firm’s innovative performance. This study also attempts to check the moderating role of perceived supervisor support (PSS) in the relationship between PCB and KH and between PCB and MD. For empirical investigation, the present study collected the data from 303 employees of various textile organizations in china through a structured questionnaire method using a convenient sampling technique. The present study applied partial least square structural equation modeling for empirical analyses using Smart PLS software. The present study revealed that a PCB does not directly influence a firm’s innovative performance. However, the results confirmed that KH negatively mediates the relationship between PCB and a firm’s innovative performance. On the other hand, results also confirmed that MD negatively mediates the relationship between PCB and a firm’s innovative performance. The finding also acknowledged that the PSS does not moderate the relationship between PCB and KH. Additionally, the findings confirmed that PSS positively moderates the relationship between PCB and moral disengagement. The present study offers important practical, theoretical, and managerial implications.

## Introduction

The firms have to be competitive in current times. The modern world has witnessed many companies working hard to keep pace with modern requirements. Firms easily attract customers with an edge over their competitors. The customers prefer the firms which own and deliver innovative services and products. Similarly, the firms which lack in providing innovative solutions are not on the wish list of customers. To keep this competitive edge, companies must seek ways to promote innovative performance at the organizational level. Several factors contribute to shaping a firm’s innovative performance. Some limiting factors also prevail, hindering a firm’s innovative performance (Wang et al., 2020).

Globalization has posed additional challenges for governmental and non-governmental firms to compete in today’s knowledge-based economy and competitive corporate settings. This condition happens due to their inability to comprehend and adapt to rapid changes in competitive market trends. Firms have recognized that knowledge management is an essential component of the process of innovation (Fallatah, 2018). In order to boost their innovative performance, these organizations prefer to combine their knowledge pool with conventional resources, procedures, and capacities in different ways. Acquisition and sharing of knowledge have significantly contributed to adding value to innovative performance. Innovative performance has become necessary for firms to achieve competitive advantages (Belawati et al., 2019).

The research on firm innovative performance (FIP) concludes that knowledge management must result in constant innovation (Pérez-Luño, 2015). Nevertheless, this research frequently misrepresents innovative performance by introducing innovative products. In comparison, both are distinct approaches (Hameed et al., 2021). New products can result from current knowledge produced inside the company or the implementation of knowledge produced and shared by other companies. The first scenario depicts innovation generation, while the latter depicts innovation adoption. The difference has not been adequately examined in the literature. It results in conflicting outcomes across research studies (Hameed et al., 2021; Leung and Sharma, 2021). Based on this assumption, what will happen if the stakeholders start to hide knowledge at the organizational level? This question is embedded with certain other factors like psychological contract breach (PCB), perceived supervisor support (PSS), and moral disengagement (MD). All these factors contribute to a firm’s innovative performance either positively or negatively. This study looks into the direct role of PCB on a firm’s innovative performance to address the contributing factors toward it. Does the second question arise what impact will PCB have on FIP? The answer to this question requires an understanding of PCB. The psychological contract is based on the rules of a reciprocal relationship between the employee and an organization (Bandyopadhyay and Srivastava, 2021).

PCB occurs when one party believes the other has failed to meet its duties or promises. Since the company possesses authority, it can impose laws that require employees to fulfill their responsibilities and commitments (Zacher and Rudolph, 2021). Therefore, the employer does not bother about PCB. In this way, researchers focus solely on the consequences of PCB as experienced by employees. Employees may notice PCB more efficiently than ever due to organizational changes like reorganizing, downsizing, and redundancies (Saleem et al., 2021). PCB has been linked to job behavior and attitudes in previous studies. These attitudes and behaviors include reduced job satisfaction, worse citizenship behavior, poor organizational commitment, and increased turnover intentions (Yang and Chao, 2016; Li and Chen, 2018). Furthermore, past research has focused chiefly on the impact of favorable feelings on the relationship between PCB and job activities. A modest volume of investigations has found a link between PCB and counterproductive job behaviors. PCB can cause absenteeism on the spur of the moment, anti-citizenship behavior, and a drop in job performance (Yang and Chao, 2016). The relationship between PCB and FIP has never been studied before. The PCB influences negative behaviors in employees, and they tend to start working with negative behaviors. This situation disturbs organizational performance (Karatepe et al., 2021). Hence, the firms’ innovative performance is also disturbed in this way. Hence, a considerable gap is posed in determining the negative factors of organizational performance. The current study tries to fill this gap by assessing the role of PCB in FIP.

This kind of relationship is usually based on social exchange in which employees experience PCB and start performing poorly. It affects the innovative performance of firms. The participants in an exchange relationship deliver tangible or intangible benefits to one another (Blau, 1964). This trading connection adheres to the reciprocity standard. The reciprocal standard states that if an individual receives favorable treatment from one side, he or she must reciprocate with favorable treatment. It also holds if one side is mistreated by the other. Put another way, when a person perceives unfavorable treatment, he or she may respond with lousy treatment or terrible behavior (Huang et al., 2016). Such destructive behaviors will contribute to compromised organizational performance. Hence, FIP will also be badly affected in this way.

Certain other factors disturb the FIP, including knowledge hiding (KH) behaviors and MD of employees. KH is a behavior in which people purposefully conceal or hide information in response to demands for information from their co-workers (Connelly et al., 2012). Researchers initially concentrated on KH among individual employees revealing that the origins of knowledge KH included not only managerial’ leadership styles and employees’ perceptions of knowledge ownership but also personal and social skepticism and workplace marginalization (Bari et al., 2020). Since organizational theory, scholars have discovered that KH exists in all kinds of organizations and significantly affects the organizations’ effectiveness (Arain et al., 2020). Therefore, researchers began to analyze the role of KH in organizational performance.

Considering its significance in FIP, no prior research has looked into its mediating effects between PCB and FIP. Instead, the research evaluated the employees’ creative performance (Liao and Chen, 2018). Therefore, to fill this gap in previous studies, current research explores the mediating role of KH between PCB and FIP. The impact of PCB on FIP requires a deep investigation of other factors which may boost or deteriorate FIP. Therefore, the MD of employees is also studied in this research as a mediator. Ethics experts have increasingly used MD theory in recent years to explain why excellent individuals participate in acts that contravene their standards of morality (Bandura, 1989). People are exploiting a range of cognitive biases, called MD, to get around their moral standards (Wang et al., 2018). Till now, thorough research has identified a connection between MD and immoral behavior at work. Furthermore, research in athletics, interpersonal, and organizational psychology suggests that MD leads to more relationally destructive acts (Huang et al., 2017). Given this evidence, it is astonishing that we only have a rudimentary conceptual knowledge of the repercussions of MD in social organizations where people must collaborate to achieve a common objective which in this study is FIP (Ogunfowora et al., 2021). The authors try to fill this gap by exploring the mediating role of MD between PCB and FIP in the current investigation. Moreover, PSS contributes to organizational performance. It is a support of supervisors toward employees to improve their task performance. This notion is based on the social learning theory, which proposes the supervisor’s role as a trainer from whom the employees get the directions for improved performance (Avotra et al., 2021).

The researchers suggest that PSS plays a role in employees’ turnover intention and task performance (Afzal et al., 2019). These scholars and others suggested its role in firms’ performance. The PSS may have a regulating role in positively shaping the FIP. The current research explores its moderating role in the relationship of PCB with FIP. The following research tends to identify the role of PCB on FIP. It probably harms FIP. Mediating factors like KH and MD are also tested in this research between PCB and FIP. Lastly, this research focuses on moderating the role of PSS on FIP. This research may prove to be a valuable check and delete contribution to the field of organizational management.

## Literature review

### Theoretical underpinning

This research is based on determining the factors which affect FIP. Traditionally, innovation has been thought of as a process of invention or discovery. A range of innovative research exists that attempts to comprehend innovative activities from the viewpoints of the firm’s resource base and knowledge base, commonly known as RBV and KBV. Firms’ capacity to utilize knowledge resources and generate innovation in terms of existing information is known as FIP (Wang et al., 2020). Nonaka (1994) claimed that organizations innovate by expanding or replacing their existing knowledge base by integrating it with new knowledge. Thus, considerably enhanced qualities of production technologies, service, marketing and management methods, and business strategies within a firm, workplace, and external environment result in FIP (Chege et al., 2020).

The conservation of resources (COR) theory provides the basis for PCB. This theory helps determine the negative impacts of PCB on FIP. Individuals aspire to collect, defend, and retain resources such as items, individual qualities, situations, and energy, that are valuable to themselves (Hobfoll, 2001). Individuals can use these resources as gateways to secure their valuable assets. Employees will be short on personal resources if they discover that PCB causes them to lose valuable resources. PCB, in other words, will deplete their valuable resource. Furthermore, according to COR theory, having enough personal resources helps in their innovative performance (Sheehan et al., 2020). These resources help them function better. Employees might use their psychological capital to develop innovative suggestions for improving the delivery of services and grievance redressal methods (Kim et al., 2018). Employees learn behaviors by witnessing the cognitive and social setups wherein they exist. This idea is supported by the SIP theory (Salancik and Pfeffer, 1978). Since everyone in this environment is subjected to a particular psychological contract, any breach will harm their attitudes and behaviors. Generally, in such an informative and social context, PCB curtails employees’ service innovation activities (Kim et al., 2018). PCB may also affect the innovative performance of firms where they work. Due to its significant impact, SIP provides theoretical support to PCB.

After a negative workplace incident, individuals’ content and mental process are influenced. This notion is supported by the affective events theory (Zhao et al., 2007). PCB is a negative organizational incident in this case. Individuals with PCB experience negative feelings and therefore cannot assess the issue reasonably. Their resources may deteriorate in these conditions. Individuals who have had their resources depleted by PCB cannot operate well or contribute to the business through innovative conduct. In short, low levels of innovative behaviors are engendered by breached views in the psychological contract with deterioration in the innovative performance (Kim et al., 2018). Hence, these kinds of PCBs also contribute to spoiling the firm’s performance. Therefore, FIP cannot be achieved when PCBs are in occurrence. The theory of knowledge management is also a contributor to current research. KH behaviors at organizational levels may mediate the adverse outcomes of PCB on FIP. So, KH gets theoretical support from knowledge management theory. Knowledge management is a relatively new concept and practice that has emerged in the modern economy, and it is both a subject and a skill for management (Ode and Ayavoo, 2020). Proper knowledge management is necessary to avoid KH from organizations to achieve FIP.

Similarly, the theory of innovation provides the basis for FIP. Researchers found that external contexts significantly impact firm-level innovation (Andreeva et al., 2021). Innovation is the product of the interaction and coordination of processes in many ways. All these coordinated processes contribute to FIP.

### Psychological contract breach and firm innovative performance

A psychological contract is founded on underlying perceptions of commitments and obligations established in a business (Rousseau, 1995). Pre-employment beliefs, the selection procedure, and after-hiring socialization all form a psychological contract. In contrast to structured contracts, these are informal and unspoken. These are based on perceptual processing and understanding others’ actions and attitudes and play a significant part (Salin and Notelaers, 2017). The impression of failing to maintain these promises is known as a PCB. The damaging aspects of a supposed PCB on work behavior and attitudes, mainly work satisfaction, in-role productivity, and turnover intentions were validated in a meta-analysis (Zhao et al., 2007).

Whenever these impressions are linked to emotional responses of wrath and rejection, PCB should have much more unfavorable consequences (Robinson and Morrison, 2000). Several investigations have found that the experience of PCB (i.e., irritation, wrath, resentment, and sense of betrayal aimed at the organization) is a crucial contributor to various unpleasant consequences (Robbins et al., 2012). Employees experiencing unpleasant feelings due to a PCB are also less likely to be committed or enthusiastic about helping the organization achieve its objectives (Rai and Agarwal, 2017). Employees limit their activities in response to a perceived contract violation, leading to lower job performance. Finally, emotions of a breach might encourage employees to seek revenge which can lead to workplace misbehavior and poor firm performance (Rai and Agarwal, 2017). A review of the literature enables identifying innovative performance in terms of the competitive position of the innovative organization, i.e., the degree to which the firm deviates from existing practices in knowledge creation and product developments or mechanisms that are effectively commercialized (Pérez-Luño, 2015). According to a prior study, if a psychological breach occurs, the organization may lose its resolve toward innovative performance due to the adverse effects on its benefits (Sharkawi et al., 2013). Therefore, the importance of exploration of the negative impact of PCB on FIP was emphasized by academics (Mohamad and Badawy, 2016). The authors developed the following hypothesis in this regard.

***H1:*** *Psychological contract breach has a negative relationship with a Firm’s Innovative Performance*

### Mediating the role of knowledge hiding

Management must put out significant effort to carry out innovation or technology invention actions to boost the firm’s innovation performance in an incentivized market competition (Rong et al., 2018). According to innovation theories, regardless of innovativeness, management must make choices based on creativity, defined by knowledge sharing or exchange, to boost the firm’s innovative capabilities (Zhang and Zhu, 2021). Furthermore, KH behavior obstructs knowledge transfer and communication among managers and employees, preventing management from sharing additional knowledge. KH behavior, according to Phillips et al. (2017), creates communication obstacles and reduces collaborative efficiency, preventing employees from effectively obtaining and using intellectual capital for innovative solutions.

As a result, it reduces the value of decision-making and impacts the company’s innovation behavior. According to Connelly et al. (2012), KH not only obstructs employees’ ability to use knowledge rationally but also causes them to be unable to create a mental map. They can use a mental map to assist them in learning what they need to know. KH may harm employees’ ability to detect and solve emergent problems promptly. In contrast, information hiding on an individual scale can fulfill the demands of other members and assist them in improving their quick performance. It is not a good idea to do so on a group level (Connelly et al., 2012). The psychological contract also includes expectations about appropriate employment climate and social norms.

Workers are encouraged to anticipate a safe working environment and to be respected and valued by their employer. Nevertheless, this assumption will probably be broken if an employee is subjected to ongoing unpleasant behavior (Salin and Notelaers, 2017). The PCB shows a relationship with the KH. Employee relationships are more likely to be harmed by evasive hiding and playing dumb than by rational hiding. According to Shen et al. (2019), PCB is linked to weaker work engagement and more significant turnover targets. Effective communication with management and other organizational authorities also strengthens the psychological contract. Following PCB, the affected employees purposefully suppress their knowledge during the staff meeting (Wang and Hsieh, 2014). This literature proposes a mediating role of KH between PCB and FIP, so the current study proposed the following.

***H2:*** *Knowledge hiding negatively mediates the relationship between psychological contract breach and a Firm’s Innovative Performance*

### Mediating role of moral disengagement

Bandura’s (1991) research on MD is based on his broader societal cognitive self-regulation theory. According to this theory, humans have self-regulatory abilities which enable them to control their thinking and behaviors. A self-regulatory mechanism that is deliberate, predictive, and constructive also guides moral thoughts and acts. Moral standards which people establish during years of socialization form the basis of the moral self-regulatory system. Such standards define the lines between what is considered moral and what is considered immoral behavior. When people consider engaging in conduct that violates their moral standards, they are less inclined to do so because they foresee psychological consequences such as remorse and contempt (Bandura, 1991). Bandura contends that MD that disables standard moral self-regulatory systems can help people avoid feeling terrible about a transgression (Bandura, 1991). Every person in the world does have a set of criteria with which he or she is content. Once these are breached, they become irritated. They overlook the terrible consequences of their actions, however, in order to avoid these feelings. MD theory provides a framework for understanding the process of rationalization of unjust conduct (Bandura, 1991). MD reduces dissonance used to rationalize unethical behavior (Bonner et al., 2016).

According to Bonner et al. (2016), the leader’s unethical behavior directly impacts the employees’ sentiments. Managers favor individuals connected to them that negatively impact other employees’ feelings and generate challenging circumstances for them, so they receive less assistance from the executives. According to Ko and Hur (2014), psychological contracts are founded on the social exchange between firms and employees. When mutual expectations are realized, employees have favorable work attitudes, but when not met, they have negative attitudes and feelings at work. Therefore, PCB strongly impacts the MD of employees, which negatively impacts the firms’ performance. Therefore, it leads to an investigation of mediating role of MD between PCB and FIP. So, the following hypothesis is proposed.

***H3:*** *Moral disengagement negatively mediates the relationship between psychological contract breach and a Firm’s Innovative Performance*

### Moderating role of perceived supervisor support

Employees’ perceptions of how supervisors provide instrumental and emotional support are referred to as PSS (Thoits, 1985). Even though the advantages of PSS are generally established, before Kelley’s (1998) publication on followership 1998, most existing research focused on a leader-centric perspective, which considered supervisors as tremendous and staff as inferior and submissive. PSS is thought to assist employees’ learning and development because it establishes supportive relationships with someone that make it easier for them to learn (Mink et al., 1993). Engaged followers, on the other hand, show independent critical thinking and a high level of job competency, necessitating less supervision (Jin et al., 2016).

Employee impressions of strong supervisor support may counteract the impact of an active support base (Jin et al., 2016). PSS is centered on social interactions between the employee and his or her boss. The social exchange theory helps to explain an employee’s loyalty to his or her boss. According to this theory, an employee’s commitment supervisor is an exchange relationship in which the employee expects to get incentives and benefits in exchange for his or her effort and dedication to the supervisor (Bandura, 1991). According to the social exchange theory, when employees gain support from their bosses, they repay by engaging in actions that benefit the supervisors (Sloan, 2012). Encouragement from supervisors and colleagues is a critical method for improving working conditions by lowering stress. Reduced stress improves job happiness, performance, and the intention to stay on the job. Prior research has shown that supervisors have a critical role in influencing employees’ perception of work environments, like influencing individual compliance with organizational procedures. Managers’ assistance varies depending on the level of support required (Puah et al., 2016). Supervisors, for example, might aid their staff by lowering their work at peak times of intense training courses and by giving training and education when needed. Nevertheless, employees’ level of trust in their supervisors significantly impacts the quality of the employer-employee relationship (Commer et al., 2015). Considering the regulating role of PSS discussed in the literature as mentioned above, this study supposed that PSS may have a moderating role in shaping the relationships between PCB, KH and FIP. So, the following are proposed.

***H4:*** *Perceived supervisor support positively moderates the relationship between psychological contract breach and knowledge hiding.*

***H5:*** *Perceived supervisor support positively moderates the relationship between psychological contract breach and moral disengagement*

**FIGURE 1:**
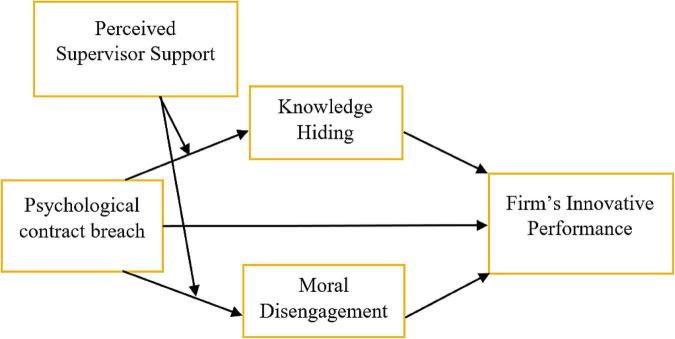
Conceptual framework.

The conceptual framework of this study is presented in Figure 1.

## Research methods

### Study design

The present study gathered data from employees of various textile sectors in China. Aibar-Guzmán et al. (2022) point out that the textile sector is largely ignored in literature. Therefore, this study considers the textile sector of data collection. This study applied a convenient sampling method for data collection (Winton and Sabol, 2021). In this regard, managers of textile organizations are contacted and a detailed meeting is conducted with them regarding the purpose of data collection. The managers were very conscious of data confidentiality; hence, it assured them that this data would be used for research purposes only. After confidentiality assurance, the managers show their consent and allow for data collection. The dual-language questionnaire was developed for the convenience of the employees. First, the questionnaire was developed in English and then translated into Chinese under the guidance of senior researchers. The sample data was collected from students to eliminate language errors and revised accordingly. The senior researchers approved the final version of the questionnaire. The questionnaire was also developed along with a cover letter. This letter conveyed the purpose of data collection to the employees by assuring them of their data confidentiality. For example, data would be gathered for purely research purposes, individual-level responses would be destroyed, and aggregated outcomes would be revealed. Moreover, the cover letter also confident the employees about the answers, such as no answers are wrong and right, and their true answers would be appropriate for this study’s true outcomes. Hence, while filling out the questionnaires, do not consult answers with your colleagues. This step ensured that getting data was as natural as possible.

A time lag data method was applied to eliminate common method bias (Bari et al., 2020; Bashir et al., 2021). For this purpose, questionnaires were distributed four times. For the first time, questionnaires related to an independent variable such as PCB were distributed; after a 1-month gap, questionnaires regarding mediating variables such as KH and MD were distributed. After a 1-month gap, questionnaires regarding dependent variables such as FIP were distributed. Lastly, the questionnaires regarding PSS were distributed after a further 1-month gap. Hence, the questionnaires included a hidden code that helped to recognize the same respondents. This study collected 468 questionnaires for the first time and 383 questionnaires for the second time. Similarly, 340 valid and complete questionnaires were collected for the third time, and finally, this study collected 303 complete and valid questionnaires for the empirical analyses. Hence, the current study is based on a 303 sample size.

### Measures

This study used five points Likert scale to measure the participants’ responses. This scale is comprised of five numbers where 1 means “strongly disagree,” 2 means “disagree,” 3 means “neutral,” 4 means “agree,” and 5 means “strongly agree.” This study considered previously validated items to assess the variables.

#### Psychological contract breach

The construct PCB is measured using a five-item scale adopted from Robinson and Morrison (2000). The sample item included, “I have not received everything promised to me in exchange for my contributions.”

#### Knowledge hiding

The variable KH is measured with six items scale adopted from Duan et al. (2022). The sample item included, “I pretended not to know or understand what he/she said, although I knew.”

#### Moral disengagement

The construct of MD was measured by adopting an eight-item scale from Moore et al. (2012). The sample item included, “People who get mistreated have usually done something to bring it on themselves.”

#### Firm’s innovative performance

A firm’s innovation performance is measured using the three-item scale of Groza et al. (2021). The sample item included, “Our business seeks new ways to do things.”

#### Perceived supervisor support

Perceived supervisor support is measured using the four-item scale adopted from Kuvaas and Dysvik (2010). The sample item included, “My work supervisor really cares about my wellbeing.”

## Results

### Assessment of measurement and structural model (mediation)

The present study applied the variance-based partial least squares structural equation modeling (PLS-SEM) technique. The key purpose behind this selection is that PLS-SEM is useful for both types of studies, including; confirmatory and exploratory (Hair et al., 2011). The covariance-based (CB-SEM) and PLS-SEM are two different and important types of structural equation modeling (SEM) (Hair et al., 2019a). The main difference in both methods is that CB-SEM is considered for theory acceptance and rejection, while PLS-SEM is applied for advancing and developing the theories (Xiaolong et al., 2021; Yingfei et al., 2021). PLS-SEM is a useful approach for complex and multi-orders-based models and needs no specific data normality conditions. PLS-SEM is also appropriate for evaluating small data sets (Nawaz et al., 2021). Hence, the present study considers the PLS-SEM method for empirical data analyses using Smart PLS 3.3.3 software. The results of PLS-SEM-based analysis are assessed in two stages, including model measurement and structural model evaluation. The measurement model stage evaluates the reliability and validity of the constructs, whereas the structural model examines the relationship between the proposed hypotheses. The acceptance or rejection of a hypothesis is determined through the “*t*” statistic and “*p*” values.

The model consists of four variables and 22 reflective indicators (Table 1). The results of model measurement consist of two parts: model reliability and validity. The present study considered the values of “Cronbach’s alpha, roh-A, composite reliability, and average variance extract (AVE)” to authenticate the model’s reliability (Hair et al., 2011), and all values are presented in Table 1. The values of Cronbach’s alpha are accepted if they are greater than 0.7 (Hair et al., 2019b). In the same way, the composite reliability value should also be greater than 0.7. The Cronbach’s alpha values of models’ constructs (firms’ innovative performance, KH, MD, and PCB) are 0.756, 0.911, 0.891, 0.885, and the composite reliability values of models’ constructs are 0.859, 0.931, 0.913, and 0.916, respectively. All values of Cronbach’s alpha and composite reliability are according to acceptable standards, which confirm the model’s reliability of the present study. The values of roh-A reliability (0.766, 0.916, 0.893, and 0.886) are also according to acceptable criteria (Hao et al., 2020; An et al., 2021). The average variance extract (AVE) values exceeding 0.5 are acceptable for the model’s convergent validity (Hair et al., 2017). Table 1 demonstrates that the AVE values (0.671, 0.692, 0.568, and 0.686) are according to acceptable criteria.

**TABLE 1:** Reliability and convergent validity of the study constructs (mediation).

Construct	Item	Outer loadings	VIF	Alpha	roh-A	Composite reliability	AVE
FIP	FIP1	0.848	1.543	0.756	0.766	0.859	0.671
	FIP2	0.783	1.444				
	FIP3	0.825	1.613				
KH	KH1	0.855	2.667	0.911	0.916	0.931	0.692
	KH2	0.801	2.309				
	KH3	0.850	2.610				
	KH4	0.835	2.345				
	KH5	0.855	2.637				
	KH6	0.794	1.921				
MD	MD1	0.805	2.872	0.891	0.893	0.913	0.568
	MD2	0.732	1.913				
	MD3	0.768	2.324				
	MD4	0.788	2.479				
	MD5	0.706	1.820				
	MD6	0.737	2.114				
	MD7	0.750	2.277				
	MD8	0.736	2.112				
PCB	PCB1	0.836	2.222	0.885	0.886	0.916	0.686
	PCB2	0.788	1.908				
	PCB3	0.842	2.358				
	PCB4	0.881	3.097				
	PCB5	0.791	1.050				

FIP, Firm Innovative Performance; KH, Knowledge Hiding; MD, Moral Disengagement; PCB, Psychological Contract Breach.

**FIGURE 2:**
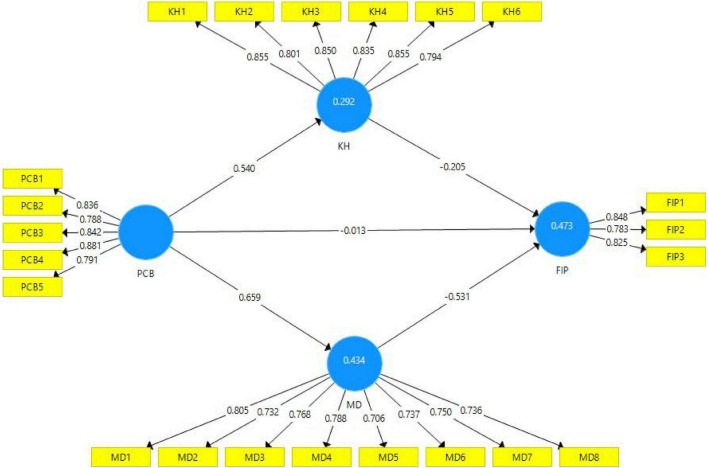
Path estimates (mediation).

All items’ outer loading values of models’ constructs are depicted in Table 1. According to experts’ given criteria, the outer loading values exceeding than or equal to 0.7 are considered reliable for the model’s validity (Hair et al., 2016). Figure 2 depicts that the outer loading values of all items are according to the acceptance criteria. The variance inflation factor (VIF) values are also depicted in Table 1. The VIF values are measured to verify the collinearity issues in the model. The model is considered free from the collinearity problems if the VIF values are below 0.5 (Hair et al., 2019b). According to the results presented in Table 1, all VIF values are less than 0.5, and the variable “PCB” item PCB-4has the highest VIF value (3.097). Hence, it is confirmed that the model of the present study is free from collinearity issues.

The *R*^2^ values are considered to define the model’s strength. The values of latent variables exceeding or near 0.5 indicate moderate strength of the model, and the values near 0.25 show weak model strength (Hair et al., 2017). The *R*^2^-values of endogenous variables of the current study’ model (KH, MD and firm’s innovative performance) are 0.292, 0.434, and 0.473, respectively, which shows moderate model strength. The *Q*^2^ (cross-validated redundancy) values of the present study’s model are considered significant if they are greater than zero (Hair et al., 2017). The *Q*^2^-values of all latent variables of the present study are according to desirable criteria, which demonstrate the significance of the model.

The present study considered two well-known approaches, namely, Fornell–Larcker criterion and heterotrait–monotrait (HTMT) ratios, to authenticate the discriminant validity of the present study (Hair et al., 2011). The Fornell-Larcker criterion is calculated by taking the square roots of AVE values of model constructs. The Fornell-Larcker criterion values of variables are presented in Table 2. The values under the Fornell-Larcker criterion are considered appropriate if the upper side first value of each column is highest than their below values. Table 2 shows that all values of the Fornell-Larcker criterion are as per the accepted criteria. Thus, it is confirmed that discriminant validity based on the Fornell-Larcker criterion has been achieved in this study model. In addition, according to the given criteria, the HTMT values of all constructs should be less than 0.85; however, values above to 0.90 are also acceptable (Hair et al., 2019a). According to the findings of the present study, the HTMT values of constructs are less than 0.85, which confirmed that discriminant validity in the present study’s model has been achieved (Table 3).

**TABLE 2:** Discriminant validity (Fornell-Larker-1981 criteria) (mediation).

Construct	FIP	KH	MD	PCB
FIP	***0.819***			
KH	–0.546	***0.835***		
MD	–0.669	0.630	***0.753***	
PCB	–0.473	0.540	0.659	***0.828***

FIP, Firm Innovative Performance; KH, Knowledge Hiding; MD, Moral Disengagement; PCB, Psychological Contract Breach.

Bold and italic values shows the significance value and relationship between variables.

**TABLE 3:** Discriminant validity (HTMT) (mediation).

Construct	FIP	KH	MD	PCB
FIP	–	–	–	–
KH	0.646	–	–	–
MD	0.807	0.693	–	–
PCB	0.573	0.592	0.733	–

FIP, Firm Innovative Performance; KH, Knowledge Hiding; MD, Moral Disengagement; PCB, Psychological Contract Breach.

### Hypotheses testing, direct and indirect relationships

The empirical investigation of this study was accompanied by using a bootstrapping approach through 5,000 samples with replacements to estimate the significance level. The direct, indirect, and total paths are presented in Table 4. The current study considered the “*t*” values and “*p*” values of statistics for the acceptance or rejection of the hypotheses. The current study hypothesis results are displayed in Table 5. According to the first hypothesis, a PCB has a negative relationship with a firm’s innovative performance; however, the outcomes (*t* = 0.191, *p* = 0.848) depicted that the PCB does not directly influence a firm’s innovative performance. Hence, the first hypothesis of this study is rejected.

**TABLE 4:** Direct, indirect and total path estimates (mediation).

	Beta	*SD*	*t*	*p*
**Direct path**				
KH - > FIP	–0.205	0.070	2.919	**0.004**
MD - > FIP	–0.531	0.080	6.606	**0.000**
PCB - > FIP	–0.013	0.066	0.191	**0.848**
PCB - > KH	0.540	0.072	7.508	**0.000**
PCB - > MD	0.659	0.056	11.738	**0.000**
**Indirect path**				
PCB - > KH - > FIP	–0.111	0.043	2.571	**0.010**
PCB - > MD - > FIP	–0.350	0.072	4.878	**0.000**
**Total path**				
KH - > FIP	–0.205	0.070	2.919	**0.004**
MD - > FIP	–0.531	0.080	6.606	**0.000**
PCB - > FIP	–0.473	0.086	5.492	**0.000**
PCB - > KH	0.540	0.072	7.508	**0.000**
PCB - > MD	0.659	0.056	11.738	**0.000**

FIP, Firm Innovative Performance; KH, Knowledge Hiding; MD, Moral Disengagement; PCB, Psychological Contract Breach.

Bold values shows the significance value and relationship between variables.

**TABLE 5:** Hypotheses testing (mediation).

Hypotheses		Coefficient (beta)	SD	*t*	*p*	Status
H1	PCB - > FIP	–0.013	0.066	0.191	0.848	Not supported

**Mediation hypotheses**		**Coefficient (beta)**	**SD**	***t***	***p***	**Status**

H2	PCB - > KH - > FIP	–0.111	0.043	2.571	0.010	Supported
H3	PCB - > MD - > FIP	–0.350	0.072	4.878	0.000	Supported

FIP, Firm Innovative Performance; KH, Knowledge Hiding; MD, Moral Disengagement; PCB, Psychological Contract Breach.

The present study also considered the mediating role of KH and MD in the relationship between PCB and a firm’s innovative performance, respectively. For the empirical investigation of KH and MD as mediators, this study assumes H2 and H3. The outcomes of H2 (*t* = 2.571, *p* = 0.010) confirm that KH negatively mediates the relationship between PCB and a firm’s innovative performance. The path value (–0.111) also confirms that the H2 of the present study is accepted. The third hypothesis results (*t* = 4.878, *p* = 0.000) confirm that MD negatively mediates the relationship between PCB and a firm’s innovative performance. The beta value (–0.350) also confirms that H3 of the present study is accepted.

### Assessment of measurement and structural model (moderation analysis)

Smart-PLS authorizes a two-stage moderation analysis method for a reflective measurement model, including model measurement and model estimation. The moderation analysis of this study depicts that all basic criteria (construct reliability and validity) and indicators of model assessment, such as “outer loading values, CR, Cronbach’s alpha, rho_A, and AVE values,” are according to acceptable criteria (Hair et al., 2016). Table 6 describes the particulars of model assessment indicators. The results of moderation analysis confirmed the discriminant validity with moderation effect (PSS) through two approaches (Fornell–Larcker criterion and HTMT ratios). Tables 7, 8 illustrate the results of the Fornell–Larcker criterion and HTMT ratios (moderations). The results also explain that the inner VIF values of all variables are significantly lower than 5 (Table 6), suggesting no collinearity issues in the present study’s data (moderation). The *R*^2^ values of endogenous variables of the current study’s model (KH, MD, and FIP) are 0.316, 0.475, and 0.473, respectively, which shows moderate model strength (Hair et al., 2019a).

**TABLE 6:** Reliability and convergent validity of the study constructs (moderation).

Construct	Item	Outer loadings	VIF	Alpha	roh-A	Composite reliability	AVE
FIP	FIP1	0.848	1.543	0.756	0.766	0.859	0.671
	FIP2	0.783	1.444				
	FIP3	0.825	1.613				
KH	KH1	0.855	2.667	0.911	0.916	0.931	0.692
	KH2	0.801	2.309				
	KH3	0.850	2.610				
	KH4	0.835	2.345				
	KH5	0.855	2.637				
	KH6	0.794	1.921				
MD	MD1	0.805	2.872	0.891	0.893	0.913	0.568
	MD2	0.732	1.913				
	MD3	0.768	2.324				
	MD4	0.788	2.479				
	MD5	0.706	1.820				
	MD6	0.737	2.114				
	MD7	0.750	2.277				
	MD8	0.736	2.112				
PCB	PCB1	0.836	2.222	0.885	0.886	0.916	0.686
	PCB2	0.788	1.908				
	PCB3	0.842	2.358				
	PCB4	0.881	3.097				
	PCB5	0.791	2.050				
PSS	PSS1	0.840	2.277	0.896	0.902	0.927	0.762
	PSS2	0.882	2.441				
	PSS3	0.914	3.427				
	PSS4	0.854	2.375				

FIP, Firm Innovative Performance; KH, Knowledge Hiding; MD, Moral Disengagement; PCB, Psychological Contract Breach.

**TABLE 7:** Discriminant validity (Fornell-Larker-1981 criteria) (moderation).

Construct	FIP	KH	MD	PCB	PSS	PSS*PCB ON KH	PSS*PCB ON MD
FIP	***0.819***						
KH	–0.546	***0.832***					
MD	–0.669	630	***0.753***				
PCB	–0.473	0.540	0.659	***0.828***			
PSS	0.023	0.127	–0.099	–0.016	***0.873***		
PSS*PCB ON KH	–0.187	0.146	0.255	0.114	0.032	***1.000***	
PSS*PCB ON MD	0.187	0.146	0.255	0.114	0.032	1.000	***1.000***

FIP, Firm Innovative Performance; KH, Knowledge Hiding; MD, Moral Disengagement; PCB, Psychological Contract Breach.

Bold and italic values shows the significance value and relationship between variables.

**TABLE 8:** Discriminant validity (HTMT) (moderation).

Construct	FIP	KH	MD	PCB	PSS	PSS*PCB ON KH	PSS*PCB ON MD
FIP	–	–	–	–	–	–	–
KH	0.646	–	–	–	–	–	–
MD	0.807	0.693	–	–	–	–	–
PCB	0.573	0.592	0.733	–	–	–	–
PSS	0.058	0.140	0.110	0.057	–	–	–
PSS*PCB ON KH	0.211	0.156	0.273	0.123	0.034	–	–
PSS*PCB ON MD	0.211	0.156	0.273	0.123	0.034	1.000	–

FIP, Firm Innovative Performance; KH, Knowledge Hiding; MD, Moral Disengagement; PCB, Psychological Contract Breach.

### Hypotheses testing (moderation)

The present study also assessed the moderating role of PSS in the relationship between PCB and KH and between PCB and MD, respectively. For empirical investigation present study assumes H4 and H5. The results (*t* = 1.064, *p* = 0.287) revealed that PSS does not moderate the relationship between PCB and KH; therefore, H4of the present study is rejected (Table 9). Additionally, the results (*t* = 3.087, *p* = 0.002) confirmed that PSS positively moderates the relationship between PCB and MD. Hence H5 of the present study is accepted.

**TABLE 9:** Hypotheses testing (moderation).

	Moderation hypotheses	Coefficient (beta)	SD	*t*	*p*	Status
H4	PSS*PCB - > KH	0.086	0.081	1.064	0.287	Not supported
H5	PSS*PCB - > MD	0.198	0.064	3.087	0.002	Supported

FIP, Firm Innovative Performance; KH, Knowledge Hiding; MD, Moral Disengagement; PCB, Psychological Contract Breach.

**FIGURE 3:**
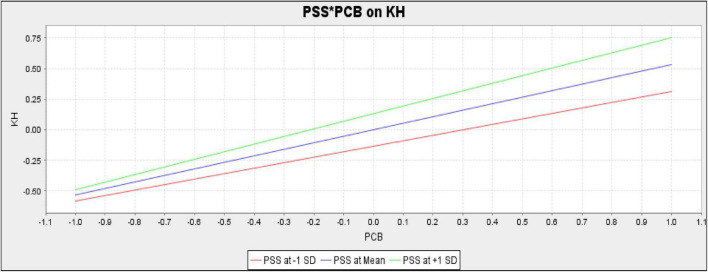
Slope for perceived supervisor support (PSS) and psychological contract breach (PCB) on knowledge hiding (KH) (moderation).

**FIGURE 4:**
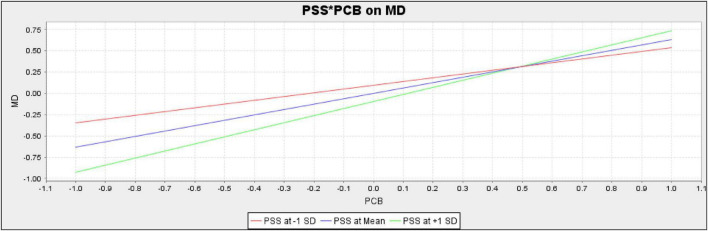
Slope for perceived supervisor support (PSS) and psychological contract breach (PCB) on moral disengagement (MD) (moderation).

The PSS does not moderate the slope for the relationship between PCB and KH. The slope is given in Figure 3. However, PSS moderates the slope for the relationship between PCB and MD. The slope is given in Figure 4.

## Discussion

Globalization paves the way for additional challenges for firms to compete in today’s knowledge-based economy and competitive corporate settings. Organizations must comprehend and adapt to rapid changes in competitive market trends in this turbulent environment. In this competitive era, firms have recognized that human capital is an important source of a competitive edge (Belawati et al., 2019). However, knowledge management is essential to taking advantage of employees’ unique knowledge and skills. To enhance innovative performance, organizations prefer to combine the unique attributes of employees with resources, procedures, and capacities in different ways. Knowledge acquisition and sharing within the firm is a major source of boosting innovative performance. Based on the social exchange theory, the present study assumes that a PCB negatively affects a firm’s innovative performance. The present study also assessed the mediating role of KH and MD in the relationship between PCB and a firm’s innovative performance. This study also attempts to check the moderating role of PSS in the relationship between PCB and KH and between PCB and MD.

The outcomes of the present study revealed that the first hypothesis is not accepted, which means that a PCB does not directly associate with a firm’s innovative performance. However, these findings are inconsistent with the prior studies (Sharkawi et al., 2013; Mohamad and Badawy, 2016). According to these studies, if employees’ PCB occurs, organizations’ innovative activities may slow down due to negative beliefs of employees about their management. Additionally, when employees believe their organizations are not fulfilling their promises, their expectations hurt, and they lose interest in the firm’s innovative activities. However, the results of present study acknowledged that the PC breach of employees has no impact on their innovative abilities. Bashir et al. (2021) highlighted the difference between a PC breach and a violation and argued that a violation of a PC is a severe condition and has adverse consequences on employees’ job performance compared to a PC breach. Therefore, the firms’ innovative performance may not be affected because the breach of PC is not as harmful as the violation of PC. Moreover, the present study’s findings confirmed that KH negatively mediates the relationship between PCB and a firm’s innovative performance. When a breach of psychological contract occurs, employees lose their trust in firms, and in turn, they hide their knowledge, as Phillips et al. (2017) point out that a PCB prevents employees from effectively obtaining and using intellectual capital for innovative solutions. The findings of hypothesis 3 revealed that MD mediates the relationship between PCB and a firm’s innovative performance. According to Ko and Hur (2014), psychological contracts are founded on the social exchange between firms and employees. When firms meet mutual expectations, employees have favorable work attitudes, but when firms violate these expectations, employees have negative attitudes and feelings at work. Therefore, PCB harms the MD of employees, which in return negatively impacts the firms’ performance.

The findings of the fourth hypothesis revealed that PSS does not moderate the relationship between PCB and KH. The rejection of this hypothesis may be due to the small sample size of this study, as it is based on 303 responses from employees. The result of H4 may not be generalizable as individuals have different values and moral standards and their responses may differ. However, these findings are inconsistent with the studies of Commer et al. (2015) and Jin et al. (2016). These authors argue that supervisors have a critical role in influencing employees’ perception of work environments, like influencing individual compliance with organizational procedures. Additionally, employees’ perception of supervisor support gave them confidence and trust that constructively impacted their work performance.

## Theoretical and practical implications

The research proves to be a significant contributor to the field of psychological management. This study shed light on PCB and its adverse impacts on a firm’s innovative performance. Firstly, it contributes to the theoretical basis of the PCB. This study is based on some theories, i.e., Social exchange theory, resource-based view, knowledge-based view, COR theory, social information processing theory, affective events theory, knowledge management theory, and innovation theory. The impact of PCB adds to the theories of RBV and COR. Once the psychological contract is breached in any organization, it leads to deteriorated resources. These resources are disturbed, and employees start behaving negatively. This situation leads to poor performance.

In this way, the performance of firms gets compromised. Current research findings add to the knowledge management theory by indicating that KH at any level of organization badly affects the FIP. Therefore, it adds to the literature by suggesting employee knowledge-sharing behaviors instead of KH. COR theory also gets strengthened by the results, which indicates that psychological contracts must be kept focused while demanding their innovative performance. It adds to the conservation of the firm’s resources. Moreover, the results strengthen the innovation theory by suggesting that organizations must avoid negative behaviors and that employees should be given due consideration to their psychological contracts. Affective events theory gets strength from the negative impact of PCB on FIP. If PCBs are controlled, then it would lead to boosted FIP. The theory of MD also gets strong support from the findings of this research. Once the employees and managers are morally disengaged, then it also badly influences the FIP. This research provides some managerial implications as well. It provides insights to the managers and executives of the firms. The executives must pay heed to the psychological contracts of the employees. They should not ignore the psychological contracts. Managers and executives should take steps toward eliminating KH behaviors. They should provide environments to the staff which encourage them to participate in morally good practices in the workplace. The employees set perceptions about supervisor support in tough times. Managers should direct the supervisors to provide proper support to the staff. It would also help in achieving FIP. Overall, this research is quite helpful for future researchers.

## Limitations

Like other social sciences studies, this study also has some limitations, which may become opportunities for scholars to conduct their studies in the future. First, the sample size of this study is small, and hence the findings may not be generalizable; future studies may increase the sample size to improve the reliability of the results. Second, the present study used time lag-based data. Future studies may consider longitudinal data to understand better the impact of PCBs on a firm’s innovative performance. Third, this study used a structured questionnaire to collect data; future studies may consider other data collection methods, such as semi-structured, open-ended, and interview methods. Fourth, this study analyzed the impact of PCB on a firm’s innovative performance, with the mediating role of KH and MD. Future studies may introduce other mediating variables such as employees’ demotivation and counterproductive work behavior to broaden the understanding of the other antecedents which impacts a firm’s innovative performance. Finally, the present study predicts the moderating role of PSS; future studies may further introduce other moderating variables like employees’ emotional intelligence to validate the present study’s findings.

## Conclusion

The PCB of employees could be a possible reason to slow down the firm’s innovative performance. Based on the social exchange theory, the present study assumes that a PCB negatively affects a firm’s innovative performance. This study also considered the mediating role of KH and MD in the relationship between PCB and a firm’s innovative performance. This study also tries to check the moderating role of PSS in the relationship between PCB and KH and between PCB and MD. The findings revealed that a PCB does not directly influence a firm’s innovative performance. However, the results confirmed that KH negatively mediates the relationship between PCB and a firm’s innovative performance. The results also confirmed that MD negatively mediates the relationship between PCB and a firm’s innovative performance. The finding also acknowledged that the PSS does not moderate the relationship between PCB and KH. Additionally, the findings confirmed that PSS positively moderates the relationship between PCB and MD.

## Data availability statement

The original contributions presented in this study are included in the article/supplementary material, further inquiries can be directed to the corresponding author.

## Ethics statement

Ethical review and approval was not required for the study on human participants in accordance with the local legislation and institutional requirements. Written informed consent from the patients/participants OR patients/participants legal guardian/next of kin was not required to participate in this study in accordance with the national legislation and the institutional requirements.

## Author contributions

LZ contributed to the conceptualization, data collection, and writing the draft and agreed to the submitted version of manuscript.
